# Redox Regulation in Amyotrophic Lateral Sclerosis

**DOI:** 10.1155/2013/408681

**Published:** 2013-02-25

**Authors:** Sonam Parakh, Damian M. Spencer, Mark A. Halloran, Kai Y. Soo, Julie D. Atkin

**Affiliations:** ^1^Department of Biochemistry, La Trobe Institute for Molecular Science, La Trobe University, Vic 3086, Australia; ^2^School of Psychological Science, La Trobe University, Vic 3086, Australia; ^3^Florey Department of Neuroscience, University of Melbourne, Parkville, Vic 3010, Australia

## Abstract

Amyotrophic lateral sclerosis (ALS) is a neurodegenerative disease that results from the death of upper and lower motor neurons. Due to a lack of effective treatment, it is imperative to understand the underlying mechanisms and processes involved in disease progression. Regulations in cellular reduction/oxidation (redox) processes are being increasingly implicated in disease. Here we discuss the possible involvement of redox dysregulation in the pathophysiology of ALS, either as a cause of cellular abnormalities or a consequence. We focus on its possible role in oxidative stress, protein misfolding, glutamate excitotoxicity, lipid peroxidation and cholesterol esterification, mitochondrial dysfunction, impaired axonal transport and neurofilament aggregation, autophagic stress, and endoplasmic reticulum (ER) stress. We also speculate that an ER chaperone protein disulphide isomerase (PDI) could play a key role in this dysregulation. PDI is essential for normal protein folding by oxidation and reduction of disulphide bonds, and hence any disruption to this process may have consequences for motor neurons. Addressing the mechanism underlying redox regulation and dysregulation may therefore help to unravel the molecular mechanism involved in ALS.

## 1. Introduction 

Cellular oxidation/reduction (redox) states regulate various aspects of cellular function and maintain homeostasis [1]. Moderate levels of reactive oxygen species/reactive nitrogen species (ROS/RNS) function as signals to promote cell proliferation, regulation, and survival [2], whereas increased levels of ROS/RNS can induce cell death [1, 2]. Under normal physiological conditions, cells maintain redox homeostasis through generation of ROS which include free radical species such as superoxide (O_2_^−^) hydroxyl radicals (OH^−^) and nonradical species such as hydrogen peroxide (H_2_O_2_); and RNS, which includes nitric oxide (NO), nitronium ion (NO_2_^+^), nitrogen dioxide (NO_2_^•^), and peroxynitrite (ONOO^−^) [3–5]. RNS are by-products of nitric oxide synthase (NOS) and NADPH oxidase [6]. Increased levels of NOS have been observed in the motor neurons of amyotrophic lateral sclerosis (ALS) patients suggesting a role of RNS in pathology [7]. Higher levels of RNS can react with other free radicals such as superoxide and undergo complex reactions to form the strong oxidant ONOO^−^ which causes cellular damage [8–10].

Cells are equipped with antioxidant systems to eliminate ROS/RNS and maintain redox homeostasis, which include enzymatic antioxidants such as superoxide dismutase (SOD), peroxidase, oxidase, catalase, and nonenzymatic oxidants such as glutathione [3, 11]. Glutaredoxin and thioredoxin are redox active molecules which undergo cysteine dependent modifications, also making them preferential targets for direct oxidation [12].

Redox regulation is a fundamental cellular process involving enzymes that maintain the appropriate environment for metabolic activities and proper functioning of the cell [13]. Normally, redox homeostasis ensures that cells respond to stressors such as oxidative or nitrative stress efficiently but when it is disturbed, neurodegeneration and apoptosis can occur [11, 14]. Neurons are particularly susceptible to degeneration via redox dysregulation as the high consumption of oxygen by the brain results in a significant production of ROS [15]. Disruption in redox regulation is implicated in the pathogenesis of neurodegeneration disorders, including ALS. Interestingly, several pathogenic mechanisms linked to ALS involve redox-sensitive proteins, such as SOD1, and proteins with active-site cysteine residues, including protein disulphide isomerase (PDI), thioredoxin, and glutathione [16–20]. These proteins contain a thiol group which is highly sensitive to changes in redox conditions [12, 21]. Even slight modulations in redox state are capable of producing neurotoxic species such as NO_2_^+^, NO_2_^•^, and ONOO^−^ [14], suggesting that redox stress could be of importance in disease [9]. 

## 2. Amyotrophic Lateral Sclerosis (ALS)

ALS, also known as Charcot's or Lou Gehrig's disease, is a fatal neurodegenerative disorder that affects the upper and lower motor neurons of the primary cortex, brainstem, and spinal cord [22, 23]. The symptoms include muscle weakness and muscle spasticity eventually resulting in paralysis [24] with ALS patients generally dying from respiratory failure within 3–5 years of diagnosis. Approximately 2 per 100,000 people worldwide are affected by ALS every year [22]. Riluzole is the only FDA-approved drug currently available for ALS. Riluzole has modest efficacy. It slows disease progression and a dose of 100 mg per day also improves limb function and muscle strength although it increases life span by an average of only 2-3 months [25, 26]. Therefore, a greater understanding of the molecular mechanisms causing ALS is important in order to develop better therapeutic solutions. 

Approximately 90% of ALS cases have no genetic association and are known as sporadic ALS (SALS). However mutations in genes such as copper/zinc superoxide dismutase (*SOD1*), fused in sarcoma (*FUS*) and TAR DNA binding protein (*TARDBP*), have also been described in SALS patients; also environmental causes such as smoking and viral infection are linked to ALS [24, 27–31]. Studies have shown higher prevalence of ALS in people with a history of trauma [32] and involvement in physical activities such as soccer has also been observed in ALS patients [33, 34]; however the exact aetiology is unknown. The remaining 10% of ALS cases, known as familial ALS (FALS), are linked to mutations in specific genes [35] including SOD1, TDP-43, FUS, vesicle associated membrane protein-B (VAPB), optineurin, alsin, and ubiquilin-2 [18, 36–43]. Recently a noncoding mutation in C9ORF72 was shown to cause the greatest proportion of FALS cases [44]. SOD1 causes 15–20% of all FALS cases and was the first described and hence most widely researched gene linked to ALS [18]. Transgenic mice overexpressing ALS-associated mutant SOD1 proteins have been used extensively as disease models [45–47]. Similar to other protein disorders, the pathological hallmark of ALS is the presence of intracellular protein inclusions [48]. Misfolded wild-type and mutant forms of SOD1, FUS, and TDP-43 [41, 49, 50] are present on the inclusions found in affected tissues of ALS patients [41, 51–53]. SALS and FALS have similar symptoms and are clinically and pathologically indistinguishable. 

Wild-type SOD1 is a highly stable homodimeric protein, explained in part by the presence of an intrasubunit disulphide bond between cysteine 57 and cysteine 146 [54]. It contains both copper and zinc ions which are essential for the catalytic activity and stability, respectively [55]. Reduction of the disulphide bond results in dissociation of the dimer and the resulting protein is highly unstable and prone to aggregation [56, 57]. 

Dysfunction in multiple cellular mechanisms is linked to ALS pathology reviewed recently by Cozzolino and coworkers [58]. Many of these events are linked to redox regulation including oxidative stress, protein misfolding and aggregation, excitotoxicity, lipid peroxidation and cholesterol esterification, mitochondrial dysfunction, impaired axonal transport and neurofilament aggregation, autophagy, and ER stress [46, 59–68]. However, there is a complex interplay between these processes and the exact aetiology of the disease is unclear. It is debatable whether redox dysregulation is a primary effect or a secondary consequence of other pathologies and the association of redox regulation and cysteine rich redox regulated proteins with these mechanisms is unclear. This paper discusses the main redox linked mechanisms which are involved in ALS and their association with redox or cysteine dependent proteins. 

## 3. Possible Redox Regulated Cellular Mechanisms Involved in ALS

### 3.1. Oxidative Stress

Oxidative stress arises when the levels of ROS/RNS exceed the amounts required for normal redox signalling. While oxidative stress has been implicated as a pathological mechanism in ALS the exact role of ROS/RNS in disease processes is unclear [9, 69]. ROS causes permanent oxidative damage to major cellular components such as proteins, DNA, lipids, and cell membranes [70–72]. ROS has been detected in the spinal cord and cerebrospinal fluid (CSF) of SALS patients [17]. Increased levels of H_2_O_2_ and oxidative damage to protein and DNA have also been observed in SOD1 transgenic mice [73]. Defects in the Rac/Nox pathway leading to redox dysregulation are also linked to SOD1^G93A^ mice [74]. Furthermore dysregulation of redox regulated-tumour protein 1, ubiquitin carboxyl-terminal hydrolase isoenzyme L1, and *α*B crystallin has been observed in transgenic SOD1^G93A^ mice [75]. 

Altered redox homeostasis regulates gene expression of transcriptional factors such as nuclear factor kappa-light-chain-enhancer of activated B cells (NF-*κ*B), activator protein 1 (AP-1), and hypoxia inducible factor 1*α* (HIF-1*α*) [76]. These transcriptional factors help in maintaining homeostasis by regulating gene expression. They have a redox regulated cysteine residue at their DNA binding site [76] which can be affected due to thiol oxidation and could be influenced by ROS [77]. A direct relation between the transcription factors and redox regulation in ALS is unknown; nevertheless dysregulation in the levels of NF-*κ*B and HIF-1*α* has been observed in SALS patients, and activation of AP-1 in mutant SOD1 expressing cells, suggesting potential involvement of redox regulation in ALS pathology [78, 79].

SOD1 and its antioxidant properties have been studied extensively from the perspective of redox regulation in ALS [80, 81]. SOD1 catalyses the conversion of superoxide into hydrogen peroxide and oxygen and it undergoes cyclic reduction and oxidation of its copper ions [82]. Initially, it was proposed that ALS mutations in SOD1 result in the loss of its ability to act as an antioxidant, but further research showed that disease is not associated with its enzymatic activity [83]. However, mutations in SOD1 could produce ONOO^−^ or OH^−^ and lower its ability to catalyse superoxide [84] by reacting with nitric oxide [85]. These intermediate products are highly unstable and have been detected with other amino acids such as tyrosine. Nitrated proteins and high levels of nitrotyrosine have been detected in the CSF of both SALS and FALS patients suggesting that posttranslational modification via free radical production is present in ALS [17, 86–88]. Oxidised wild-type SOD1 in the lymphoblasts of SALS patients associates with mitochondrial Bcl-2 which causes mitochondrial damage [89]. Oxidative damage is an important phenomenon; however, treatment with antioxidants has not been very successful [90].

### 3.2. Protein Aggregation and Misfolding

Redox dysregulation may not only increase the production of ROS/RNS but also affect protein conformation and structure. Posttranslational modification of SOD1 such as oxidation has an adverse effect on the conformational arrangement of SOD1 [91]. Glutathionylation, a posttranslational modification of the 111 cysteine residue, causes destabilisation of SOD1 structure [92]. Wild-type SOD1 has been shown in inclusions of SALS patients suggesting its involvement in causing neurotoxicity [93]. Evidence suggests that oxidised wild-type SOD1 has the ability to misfold and form aggregates and gain similar conformation as the mutant and has toxic functions *in vitro* [89, 94]. SOD1 depleted zinc and copper have altered redox activity and are more prone to oxidation [95].

An oxidising environment also causes abnormal disulphide linkages and protein aggregation in ALS [80, 96]. SOD1 containing aberrant disulphide bonds involves the normally unpaired cysteine residues cysteine 6 and cysteine 111 in the spinal cord of ALS transgenic mice models [96]. Studies show that mutant TDP-43 aggregation is caused due to increased disulphide bonds [97]. Similarly oxidative stress causes aberrant disulphide cross-linking and subcellular localisation of TDP-43 [97] as well as accumulation of FUS into the cytoplasm [98]. Mutant SOD1 readily forms monomers, oligomers, or inclusions which are insoluble [55]. It is unclear how conformational changes cause misfolding but one possible explanation could be the modification and alteration of protein structure by ROS through oxidisation of the thiol group, forming aberrant disulphide bonds. 

### 3.3. Glutamate Excitotoxicity

The levels of glutamate present in mammalian CNS are much higher than those of other neurotransmitters (5–10 mmol/kg) indicating the importance of glutamate in neuronal function [99]. However, excitotoxicity occurs when the levels of glutamate are increased in neurons, resulting in increased calcium intake and neuronal injury [100, 101]. Motor neurons are particularly susceptible to high levels of glutamate [102]. Glutamate uptake from the synapse is controlled by glutamate transporters astroglial GLAST, GLT1, and neuronal EAAC1 which possess a redox regulated cysteine residue [103]. N-methyl-D aspartic acid (NMDA) glutamate receptors are also redox regulated suggesting that redox dysfunction may further affect glutamate regulation. Increased levels of intracellular glutamate and decreased uptake of glutamate from the synapse have been observed in ALS patients [104, 105]. Indeed, Rothstein and coworkers showed an absence of GLT1 transporter in ALS patients [106]. ROS can reduce the uptake of glutamate in mammals [107]; however, increased calcium levels in the mitochondria due to dysfunctional glutamate regulation can result in overproduction of ROS and cause oxidative stress [108]. The question remains whether oxidative stress causes glutamate dysregulation or vice versa.

### 3.4. Lipid Peroxidation and Cholesterol Esterification

The ER is also the main site of lipid and sterol synthesis [109]. Lipids are major targets of oxidative stress, resulting in lipid peroxidation via a chain-reaction process [11]. Sphingolipids are localised in the plasma membrane and ER membranes and, with cholesterol, are processed into domains known as lipid rafts [68]. Lipid rafts can form macroplatforms for redox signalling, providing critical mediation for cellular functioning [110]. Lipid peroxidation and cholesterol esterification have been implicated in the pathogenesis of ALS [68, 69, 111]. Excitotoxicity and oxidative stress alter sphingolipid metabolism resulting in the accumulation of long-chain ceramides, sphingomyelin, and cholesterol esters in the spinal cords of ALS patients and Cu/Zn SOD1 mice. This occurs at the early presymptomatic stage of disease in the SOD1 mice [68] thus implicating aberrant lipid metabolism in the pathophysiology of ALS. Further evidence of lipid dysregulation in ALS comes from studies which reported that ALS patients demonstrated a tendency towards hyperlipidemia. Additionally, correlational studies have shown that ALS patients with the highest low density lipoprotein (LDL)/ high density lipoprotein (HDL) ratio have a significant increase in survival time and respiratory function [112, 113]. Furthermore, recently, an interaction between SOD1 aggregates with lipid was found to alter lipid membrane permeability [114]. 

Lipid peroxidation products such as 4-hydroxynonenal have been detected at higher levels in ALS patients spinal cord than controls, and this has been linked to modification of astrocytic glutamate transporter EAAT2 and excitotoxicity [111]. Excitotoxicity was also linked to upregulation of sterol regulatory binding element 1 (SREBP1) in the spinal cords of FALS and SALS patients, and SOD1^G93A^ transgenic mice suggesting cholesterol depletion [115]. Furthermore, the link between ALS and statins, a class of drug which inhibit 3-hydroxy-3-methylglutaryl coenzyme A (HMG-CoA) reductase, may suggest that suppressing cholesterol synthesis increases the incidence [116, 117], progression, and severity of ALS [118], although this has been questioned [119]. Lipid raft alteration has also been linked to the pathogenesis of ALS. Endogenous, wild-type and mutant SOD1^G93A^ proteins were recruited into lipid rafts isolated from spinal cords of transgenic SOD1 mice [120]. Hence, together the data suggest that oxidative stress may alter sphingolipid and cholesterol metabolism and deregulate lipid raft redox signalling leading to the accumulation of toxic ceramides and cholesterol esters which may ultimately result in motor neuron death [68].

### 3.5. Mitochondrial Dysfunction

Mitochondria are important players in redox regulation and oxidative stress has the potential to cause mitochondrial dysfunction [70, 121]. Indeed, damaged mitochondria are observed in the spinal cord cells of SALS patients [122–124]. The mitochondrial genome is particularly susceptible to oxidative damage [125], hence any increase in cellular ROS would potentially perturb mitochondrial functions. Mitochondria participate in neuronal apoptotic signalling pathways through the release of mitochondrial proteins including cytochrome c into the cytoplasm [126]. There is substantial evidence that molecular components of mitochondrial apoptosis play a role in neurodegeneration in both SOD1 rodents and in mutant SOD1 overexpressed in cell culture [127]. The enzymatic activity of cytochrome c oxidase (COX) in mitochondria is also reduced in the spinal cord cells of SALS patients [122–124, 128, 129]. Mitochondria have been well studied in relation to ALS pathogenesis. Degenerating or abnormal mitochondria have been described in mouse models [62, 130], cultured neuronal cellular models [131, 132], and ALS patients [133, 134], although how nonfunctioning mitochondria relate to ALS is unclear. Possible explanations include inhibition of axonal transport, dysregulation of calcium buffering [135], or activation of mitochondrial-dependent apoptosis [128, 136]. Recent studies have shown that overexpression of TDP-43 causes mitochondrial dysfunction and induces mitophagy in cell culture [137]. The presence of ROS and impairment of the mitochondrial respiratory chain have also been observed in TDP-43 models [138, 139]. 

Mutant SOD1 has also been implicated in mitochondrial respiratory complex impairment [140] and a shift in the redox state of mitochondria towards oxidation [141]. How SOD1 functions in the mitochondria is still not clear, although some data suggests that SOD1 is crucial for maintenance of the mitochondrial redox state [142, 143] and that ALS mutations affect the localisation or function of SOD1 in mitochondria [135]. However, mutant misfolded SOD1 has been found localised with various compartments of the mitochondria [144]. Significantly, any pathological changes in regulation of the electron transport chain would result in more oxidative stress [145] triggering further cellular redox dysregulation, leading to a potential vicious cycle of damage and degeneration.

### 3.6. Impaired Axonal Transport

Axonal transport is a key mechanism required for cellular viability in neuronal cells. Most proteins required in the axon and in synaptic terminals must be transported along the axon after synthesis in the cell body. Similarly RNA and organelles also need to be transported over long distances, and these transport processes require molecular motors, such as kinesins, dyneins, and myosins that operate along the cellular cytoskeleton. Dysfunction of axonal transport has now been well documented in ALS [61]. Whilst many of these studies implicate dynein in this process [146], several also highlight the importance of kinesin in ALS, particularly kinesin heavy chains KIF5A and KIF1B*β*, which transport mitochondria, synaptic vesicles, and macromolecular complexes. Interestingly, a recent study demonstrated that oxidised wild-type SOD1 immuno-purified from SALS patient tissues inhibited kinesin-based axonal transport in a manner similar to mutant SOD1 in FALS providing evidence for common pathogenic mechanisms in both SALS and FALS [94]. 

Neurofilaments (NF) accumulation in motor neurons is another histopathological hallmark of ALS [147, 148]. Also, transgenic mice that overexpress NF subunits in motor neurons develop a motor neuron disease with impaired axonal flow, as axonal defects cause delay in transportation of components required for the maintenance of axon [149]. However, ONOO^−^ formed during oxidative stress from nitrooxide and superoxide can affect NF assembly and cause NF accumulation in motor neurons [8]. Chou and coworkers showed NF aggregations are associated with SOD1 and nitric oxide synthase activities leading to nitrotyrosine formation on NF [150]. Nitrotyrosine can inhibit phosphorylation of heavy or light NF subunits and may alter axonal transport and trigger motor neuron death [150]. Taken together, these findings suggest a relation between redox regulation and axonal transport dysfunctions in ALS.

### 3.7. Autophagy

Autophagy is a normal homeostatic mechanism to dispose large protein aggregates, damaged organelles, and long-lived proteins. Autophagic stress results when the number of autophagosomes increases relative to the proportion of degradable proteins. The presence of high levels of superoxide and hydrogen peroxide species can induce autophagy *in vitro* [151], but consequently, autophagy can further induce oxidative or nitrative stress thus creating a vicious cycle [152]. Dysregulated redox activity also influences autophagy. Cathepsin, a class of proteases which have highly regulated thiol groups [152] and other key regulatory autophagic complexes such as Beclin 1 and Rubicon, also have the presence of cysteine residues [152]. The presence of cysteine residues suggests that they are redox regulated and likely to be affected by ROS. ATG 4, another protease, is a target of oxidation by hydrogen peroxide. However, direct association of these with ALS has not yet been identified. Altered autophagic levels have been observed in SOD1^G93A^ mice and sporadic and familial patients but whether the increased levels are protective or not is still questionable [153–156].

### 3.8. ER Stress and Protein Disulphide Isomerase (PDI) in ALS

The ER is redox regulated and another important location for the production of ROS. It plays key roles in protein and lipid synthesis and protein folding. Protein misfolding within the ER triggers ER stress which induces the unfolded protein response (UPR) a distinct signalling pathway which aims to relieve stress [157]. While initially protective, prolonged UPR causes apoptosis [158, 159]. Recent studies suggest that ER stress is an early and important pathogenic mechanism in ALS [66, 158, 160]. ER stress is induced in animal models of SOD1, in cells expressing mutant FUS and in patients [20, 161]. Oxidative stress driven by changes in fatty acid composition, mitochondrial function, and/or proteosome activity leads to oxidative stress and contributes to ER stress in SALS patients [162, 163]. PDI is an ER chaperone which is induced during UPR and has been implicated in several neurodegenerative disorders including ALS [164–166].

**Figure 1:**
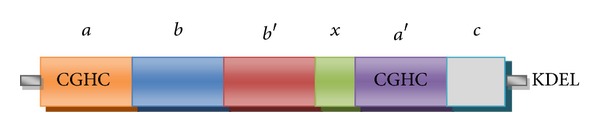
Schematic diagram showing domain structure of PDI. Thioredoxin-like *a* domain (orange) and *a*′ domain (purple) possessing the catalytic motif, catalytically inactive *b* domain (blue), and *b*′ domain (red). Green represents the linker region *x* which allows flexibility between domains. The C terminal domain is shown in grey followed by the ER retrieval signal KDEL.

PDI is a member of an extended family of foldases and chaperones which are responsible for the formation and isomerisation of protein disulphide bonds [167]. The PDI family comprises 21 members which have structural similarities but different functions [168] and all have a similar active site to thioredoxin [169]. Thioredoxin is an intracellular protein which regulates redox conditions and which is effective against oxidative stress [170]. PDI is most abundant in the ER but it is also found in other subcellular locations such as the nucleus and extracellular matrix [171] and it constitutes 0.8% of the total cellular protein [172]. The yeast PDI crystal structure was recently solved [173] which suggests that *a* and *a*′ domains are responsible for the formation of disulphide bonds (Figure 1). These domains contain a redox active CGHC motif which isomerases protein disulphide bonds and is involved in redox regulation [173]. PDI also contains *b* and *b*′ domains which are responsible for substrate binding [174, 175]. Misfolded proteins attach to the hydrophobic region of an inverted U shape structure [173, 176]. The C-terminal region also aids in polypeptide binding and contributes chaperone activity [177]. Compared to other family members, PDI has broad substrate specificities and can interact with glycosylated as well as nonglycosylated proteins [178]. 

## 4. PDI and Redox Regulation

PDI forms protein disulphide bonds by the oxidation of thiols within the PDI active site cysteine residues [179, 180]. When PDI is in an oxidised state it transfers a disulphide to the substrates thereby oxidising the substrate and becoming reduced itself. Conversely, substrates which need disulphide bond rearrangement are reduced by PDI in the reduced state thus oxidising PDI in the process [168, 181]. This continual cycling regulates redox conditions within the ER. A thiol containing tripeptide protein and glutathione also maintains ER redox homeostasis by similar shuffling between oxidized and reduced cysteine residues. Glutathione is also required for the isomerisation and rearrangement of disulphide bonds [182]. The redox potential of PDI (−110 mV) is lower than other family members [183] due to intervening residues present between the reactive cysteines thus facilitating disulphide bonds [183]. ERO1 oxidises PDI also aiding disulphide bond formations [184], but PDI is also oxidised through peroxiredoxin 4, vitamin K, glutathione peroxidase, and quiescin sulfhydryl oxidase [181]. During ER stress high levels of ERO1 have been observed which accelerates protein oxidation suggesting interplay between oxidative stress and ER stress. The transfer of electrons from the thiol group of PDI to ERO1 results in the production of excess ROS, decreasing the levels of glutathione available for reduction and increasing ERO1 thus altering the redox conditions [185, 186]. Hence, imbalance in the redox state of the ER may result in dysregulation of thiol containing proteins and triggers. 

### 4.1. The Role of PDI in ALS

Due to its function in preventing protein misfolding, PDI is important in protein quality control [166]; also deletion of PDI is embryonically lethal [187]. Hence, regulated expression of PDI is critical for normal cellular function. There is now growing evidence for a role of PDI in ALS. PDI levels are upregulated in transgenic models of ALS and spinal cord tissues of ALS patients [66, 158]. Overexpression of PDI is also protective against mutant SOD1 mediated aggregation and reduces cell death *in vitro* [20]. PDI coimmunoprecipitates with both SOD1 and FUS [158, 161]; it also colocalises with SOD1, TDP-43, and FUS in ALS patients suggesting a physical interaction exists between PDI and other key misfolded proteins in ALS [66, 161, 188]. Similarly, PDI also colocalises with TDP-43 in ALS tissues and with VAPB inclusions in a *Drosophila melanogaster* model of ALS [188, 189]. A small mimic of the active site of PDI, dithiol (±)-trans-1,2-bis (mercaptoacetamido) cyclohexane (BMC), is also protective in cell culture and it reduces mutant SOD1 aggregation in a dose dependent manner [20]. Further evidences for a role for disulphide interchange activity in ALS comes from studies showing that another PDI family member ERp57 is also upregulated in transgenic SOD1 mice and ALS patients [66]. Furthermore, thioredoxin is also upregulated in the erythrocytes of FALS patients [19]. 

**Figure 2:**
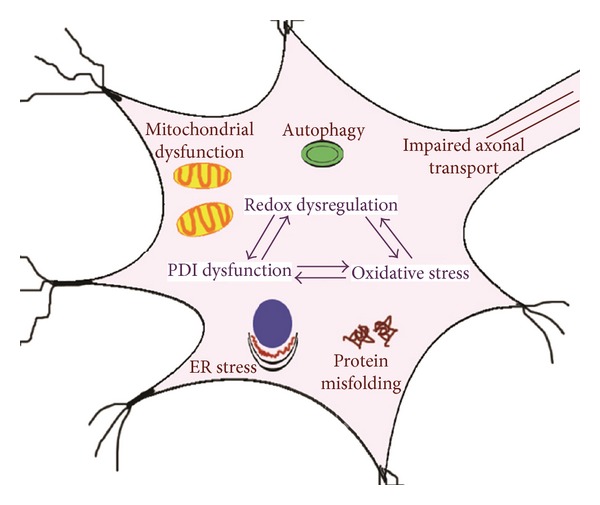
Redox dysfunction and its relationship to other pathologies in ALS. Alteration in the enzymatic activity of PDI due to redox dysregulation and oxidative stress can further increase the load of misfolded proteins, ER stress, oxidative stress, autophagy, mitochondrial dysfunction, and axonal impairment leading to neuronal cell death.

The upregulation of these thiol containing proteins in ALS suggests a cellular defensive mechanism is triggered in disease as a defence against oxidative stress. However, there is evidence that normal protective function of PDI is inhibited in disease [20]. Modifications of active site thiol groups through direct oxidation, S-glutathiolation and S-nitrosylation, can lead to inactivation of the normal enzymatic activity of PDI [13, 190, 191]. PDI was recently shown to be S-nitrosylated in ALS [20, 192] as in other neurodegenerative disorders such as Parkinson's and Alzheimer's disease. [191]. S-nitrosylation occurs when there is an increased production of RNS during oxidative stress resulting in addition of a nitrogen monoxide group to the thiol side of PDI [20, 164]. Experiments performed by Chen and coworkers suggested that in the presence of S-nitrosylated PDI, the formation of mutant SOD1 aggregates increases *in vitro* [192]. It is also likely that inactivation of PDI could lead to activation of the UPR as observed in other neurodegenerative disorders [191]. The loss of PDI functional activity can directly lead to apoptosis, or indirectly to a range of cellular abnormalities such as oxidative stress and protein misfolding, which again lead to cell death [164, 166]. Hence the redox regulation of PDI is a crucial component in the maintenance of a balanced redox environment, and inhibition of its enzymatic activity will lead to important consequences for the cell (Figure 2).

Neurons are highly susceptible to redox dysregulation due to their high metabolic requirements, large size, and lower ability to maintain the balance between antioxidants and ROS [15]. In disease states such as ALS, oxidative stress, and altered enzymatic activity of PDI, which normally reduces ROS and the burden of misfolded protein, can cause serious damage to the neuron. Since multiple mechanisms are involved in neurodegeneration, any imbalance in redox regulation can lead to an imbalance in the production of free radical species, which consequently cause mitochondrial damage and excitotoxicity, thus elevating the levels of free radicals [193]. Furthermore, an excess of free radicals can also lead to DNA damage and may also result in aggregation of NF [194] and structural destabilization of other proteins, thus inducing ER stress and apoptosis. Since ALS is a slow progressive disorder it could be hypothesised that these cyclic events, due to loss of functional activity of PDI, may gradually lead to neuronal degradation. In such a scenario, the redox regulatory function of PDI may therefore have an important protective effect. 

## 5. Conclusion

Redox regulation is an important mechanism of homeostasis in eukaryotic cells, especially neuronal cells where oxygen levels are high [15]. Many cellular processes rely on it, including proper functioning of the mitochondria and ER, calcium regulation, axonal transport, regulated autophagy, and protein folding. Links between redox dysregulation and ALS are becoming well documented in the literature, although the directionality of these links and their underlying cause are still quite unknown. One possible key player in redox regulation in ALS is PDI, whose role in ALS pathogenesis is the topic of much new research. As the critical protein involved in thiol reduction, any dysregulation of PDI activity can lead to oxidative stress and redox dysregulation. Due to its activity, PDI itself also contains an active site thiol group suggesting that it can also be affected by oxidative stress, leading to an escalating cycle that perpetuates redox dysregulation. How PDI becomes nonfunctional in the first place is still unclear, although some papers point to S-nitrosylation as having a role [20]. Regardless of its exact role, any mechanism to improve the catalytic activity of PDI should have a reductive effect on oxidative stress levels in neurons. It is therefore tempting to speculate about PDI as a possible therapeutic target in the treatment of ALS.
